# Early diagnosis of keratoconus using corneal biomechanics and OCT derived technologies

**DOI:** 10.1186/s40662-025-00435-3

**Published:** 2025-05-12

**Authors:** Xiaorui Wang, Sayo Maeno, Yixin Wang, Shizuka Koh, Shihao Chen, Andrew J. Quantock, Siân R. Morgan, Sally Hayes, Colm McAlinden

**Affiliations:** 1 Structural Biophysics Group, School of Optometry and Vision Sciences, Cardiff University, Cardiff, Wales UK; 2 the First Affiliated Hospital of Northwest University, Xi’an, Shaanxi China; 3 Department of Ophthalmology, Osaka University Graduate School of Medicine, Suita, Osaka Japan; 4 Wenzhou Medical University, Wenzhou, China; 5 Eye & ENT Hospital of Fudan University, Shanghai, China; 6 School of Optometry and Vision Sciences, Cardiff University, Cardiff, Wales UK

**Keywords:** Keratoconus, Early stage, Corneal tomography, Corneal topography, Corneal biomechanics, OCT

## Abstract

**Background:**

Early detection of keratoconus is essential for maximizing the potential of cross-linking treatments designed to halt keratoconus progression, minimizing the risks of iatrogenic ectasia as well as reducing the need for corneal transplantation. This review focuses on the progress that has been made in the early detection of keratoconus using biomechanical and topographical properties derived from three different technologies, namely the ocular response analyser (ORA), corneal visualization Scheimpflug tonometer (Corvis ST) and optical coherence tomography (OCT).

**Method:**

A PubMed search was performed using the keywords of ‘early keratoconus’, ‘subclinical keratoconus’, ‘forme fruste keratoconus’, ‘very asymmetric ectasia with normal topography/tomography’ and ‘ocular response analyser’ and/or ‘Corvis ST’/‘corneal visualized Scheimpflug tomographer/tomography’ and/or ‘optical coherence tomography/tomographer’.

**Results:**

The integration of biomechanical parameters and corneal morphological data from the topography/tomography or OCT, or the assessment of bilateral asymmetry, has demonstrated improvement in the accuracy of diagnosing early-stage keratoconus.

**Conclusions:**

As measurement principles differ depending on the technique used for keratoconus assessment, comprehensive metrics may be needed to reflect subtle anterior or posterior corneal changes and help identify eyes with very early ectasia. Although clinical experts have always, and will most likely, continue to play a pivotal role in decision-making for early keratoconus diagnosis, future developments in technology and AI may lead to enhanced early detection in the future.

## Background

Keratoconus is a bilateral condition [1], characterized by corneal elevation, reduced corneal thickness, corneal biomechanical weakening, irregular astigmatism and progressive vision loss [2]. The associated risk factors include eye rubbing, a family history of keratoconus, allergy, asthma, and eczema [3]. The early detection of keratoconus can result in a better long-term prognosis, and it is crucial that keratoconus is excluded prior to refractive surgery [4]. Performing laser ablation surgery on these candidates could result in severe progressive iatrogenic ectasia [5, 6]. The advent of corneal topography and tomography has improved the early diagnostic ability of clinicians in identifying corneal ectasia, which may correspond to the higher keratoconus prevalence rates reported in recent years; up to 1.38 per 1000 population [3].

Nevertheless, it is still a challenge to differentiate borderline cases of keratoconus from normal corneas using tomography and topography, especially in small corneas where the 8 mm diameter best-fit sphere is incompatible with the size of the cornea and can produce a falsely elevated posterior surface [7]. This is especially poignant when considering different ethnic groups, as corneal diameter has been shown to be 0.2–0.5 mm narrower in Asians versus whites [7]. A further complication is that some patients with keratoconus may present typical clinical symptoms in one eye only, with the fellow eye showing no sign of abnormalities in corneal topography/tomography or slit-lamp examinations; this is referred to as forme fruste keratoconus (FFKC). In such cases, biomechanical abnormalities may be observed despite the appearance of normal tomography and topography [8]. According to the Global Consensus on Keratoconus and Ectatic Corneal Diseases [1], diagnosing early or subclinical keratoconus involves observing posterior corneal elevation abnormalities. This is supported by a number of studies that have highlighted the prime importance of posterior corneal changes [9], and the limited ability of detecting early keratoconus with simple Placido disc topography and keratometry which does not incorporate posterior corneal changes [10]. However, studies focusing on higher-order aberrations suggest that the primary contribution comes from the anterior corneal surface for detecting subclinical keratoconus [9, 11–13], and changes in the densitometry in the anterior central zone could be useful in detecting early-stage keratoconus as well [14, 15]. Therefore, whether the initial signs of keratoconus in the early stages can be identified at the anterior or the posterior corneal surface remains controversial.

Terminology has been problematic in this area. For example, the term ‘early-stage keratoconus’ may encompass FFKC or very asymmetric ectasia (VAE) [where one eye has clinical keratoconus and the fellow eye appears topographically normal (VAE-NT)], subclinical keratoconus (SKC), keratoconus suspect, pre-keratoconus, or mild keratoconus. In this review, we have used the most common definitions of SKC and FFKC. SKC is defined as ‘an eye with topographic signs of keratoconus and/or suspicious topographic findings under normal slit-lamp examination and keratoconus in the fellow eye, whilst FFKC is defined as ‘an eye with normal topography, normal slit-lamp examination, and keratoconus in the fellow eye’ [16, 17]. This definition would suggest that FFKC is less progressed than SKC towards KC. However, in a survey of 33 studies on SKC and 22 studies on FFKC, it was not possible to identify any specific examination that clearly differentiated SKC from FFKC [16]. For instance, keratoconus percentage (KISA%) values between 60% and 100% accounted for approximately 9% in both SKC and FFKC. On the other hand, all studies reported high incidences of keratoconus in the fellow eye, i.e., 72.72% in SKC and 77.27% in FFKC as a diagnostic criterion, indicating that a bilateral early diagnosis of the disease without clinical expression is a challenge [16]. In recent years, the term VAE-NT has been used synonymously with FFKC to describe patients with defined clinical ectasia in one eye and normal topography/tomography in the fellow eye [18].

This review focuses on the progress that has been made in the early detection of keratoconus (FFKC, VAE-NT, SKC) using biomechanical and topographical properties derived from three different technologies, namely the ocular response analyser (ORA), corneal visualization Scheimpflug tonometer (Corvis ST) and optical coherence tomography (OCT). A comprehensive search was performed in PubMed using the keywords of ‘early keratoconus’, ‘subclinical keratoconus’, ‘forme fruste keratoconus’, ‘very asymmetric ectasia with normal topography/tomography’ and ‘ocular response analyser’ and/or ‘Corvis ST’/‘corneal visualized Scheimpflug tomographer/tomography’ and/or ‘optical coherence tomography/tomographer’. Only those papers which clearly defined and characterised their populations of early keratoconus with slit-lamp findings, corneal topography and/or corneal tomography properties were included in the review.

## Main text

## Ocular response analyser (ORA)

**Fig. 1:**
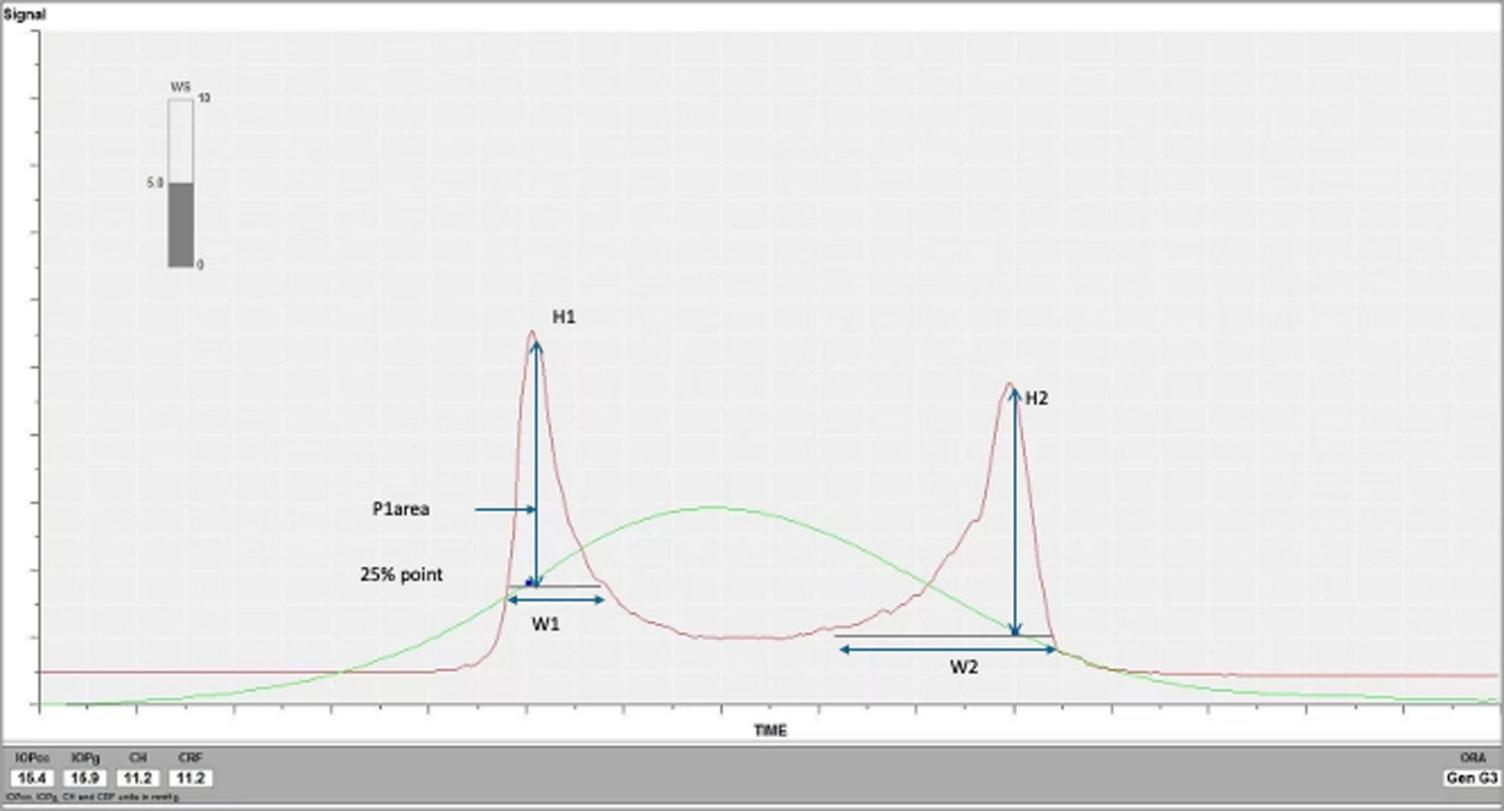
Illustration of peaks measured with the ocular response analyser in a myopic eye. CRF, corneal resistance factor; CH, corneal hysteresis; p1area, upper 75% area of first peak; H1, height of first peak; H2, height of second peak; W1, width of peak 1 at point of the 25% of the base region W2, width of peak 2 at point of 25% of the base region

The first in vivo measurements of corneal biomechanical response came with the introduction of the ORA (Reichert Ophthalmic Instruments, Buffalo, NY, USA) in 2005 [19]. The system was designed to improve the accuracy of intraocular pressure measurements by taking into account central corneal thickness (CCT) and the biomechanical properties of the tissue [19, 20]. The ORA is a noncontact tonometer which uses a collimated rapid air puff to indent the central 3–6 mm of the cornea and an advanced electro-optical system to monitor the bi-directional movement of the cornea in response to the air puff (Fig. 1). Using a reflected infrared signal, the system records the pressure as the cornea reaches its first applanation point during indentation, and then again, as it reaches its second applanation point whilst returning to its original shape [19, 21]. The difference between these two applanation pressures is thought to be caused by the viscoelasticity of the cornea and provides an estimated measure of corneal hysteresis (CH), which is an assessment of the cornea’s ability to absorb and dissipate energy or force. The ORA software also records a corneal resistance factor (CRF). Whilst CH principally relates to the viscous properties of the cornea, CRF is thought to be dominated by the elastic properties of the tissue [22].

Schweitzer et al. demonstrated that the ORA could provide additional information to assist with the screening of FFKC. Compared with normal eyes, FFKC eyes showed significantly lower values of CH (9.1 ± 1.8 vs. 10.3 ± 1.9 mmHg) and CRF (9.2 ± 1.8 vs. 11.1 ± 2.0 mmHg). Further, the force and time needed to reach applanation was significantly lower in eyes with FFKC [23]. Whilst Ayar et al. reported similar results, showing differences between FFKC eyes and normal eyes in both CH (8.3 ± 1.6 vs. 9.8 ± 1.6 mmHg) and CRF (7.8 ± 1.2 vs*.* 9.9 ± 1.5 mmHg) [24], others have reported differences in the mean CRF between FFKC and normal eyes (7.8 ± 1.4 vs. 10.2 ± 1.7 mmHg) without any statistical difference in the mean CH [25, 26]. The reason for the difference between studies is most likely due to differences in the classification of the groups, as summarized in Table 1.

**Table 1:** Previous studies on early keratoconus with ocular response analyser (ORA)

Authors	Country	Age (years)	Male (%)	Cases, eyes	Definition of FFKC/SKC/VAE-NT				Main results
					Slit-lamp examination	Topography	Others	Fellow eye	
Schweitzer et al. [23]	France	31.1 ± 9.9	58	FFKC (55, 55)	No signs of KC	KISA% < 60% (Orbscan topography)	CDVA of 20/20	KISA% index > 100%*	↓ CH and CRF in FFKC
Ayar et al. [24]	Turkey	26.5 ± 7.2	59	FFKC (27, 27)	No signs of KC	No topography (Pentacam) findings	–	Clinical KC	↓ CH and CRF in FFKC AUROC of CH (0.768) and CRF (0.866)
Zhang et al. [25]	US	47.3 ± 17.1	67	FFKC (47, 64)	No signs of KC	Keratoconus Severity Score (KSS) < 3^†^	–	–	↓ CRF in FFKC; no difference in the mean CH
Mohammadpour et al. [26]	Iran	27.6 ± 4.3	50	FFKC (21, 32)	No signs of KC	Minor topographic asymmetry, steep K > 47.0 D and oblique cylinder > 1.5 D	–	–	No difference in the mean CH or CRF after controlling for CCT and sex
Kirgiz et al. [27]	Turkey	–	–	FFKC (50, 50)	No signs of KC	Anterior asymmetry index > 0.50 and/or a posterior asymmetry index > 0.30 in combination with corneal thinning), mild steepening on topography	–	–	AUROC of CH (0.85) and CRF (0.90) for discriminating between FFKC and normal eyes
Luz et al. [28]	Brazil	25.5 ± 7.2	–	FFKC (21, 21)	No signs of KC	KISA% < 60% (Placido-disk topography) without a suspect pattern	–	Clinical KC	BAD-D (AUROC 0.91 ± 0.057), the upper 50% area of the first peak (p1area1) (AUROC 0.717 ± 0.065) A combination of topography and biomechanical parameters showed the best result (AUROC 0.953 ± 0.024)
Sedaghat et al. [29]	Iran	26.2 ± 4.3	42	ACE-NT (128, 128)	No signs of KC	Normal topography/tomography (Pentacam)	–	Clinical KC	CRF (0.866) had a higher detection ability (cut-off ≤ 9.6) than CH (0.826) (cut-off ≤ 9.9)
Kirwan et al. [30]	Ireland	36.7 ± 12.8	–	FFKC (18, 30)	–	FFKC: principal criteria were a > 1.5 D discrepancy between superior and inferior corneal curvature	–	–	CH and CRF values overlapped between the 3 groups. No difference in CH and CRF measurement between FFKC and normal eyes
Fraenkel et al. [31]	Germany	33.6 ± 13.3	65	VAE-NT (26, 26)	No slit-lamp findings	Normal topography including the posterior corneal surface with a change in elevation (posterior elevation difference) (from the baseline to the exclusion map) of 12 μm or less (back elevation map and BAD-D)	–	Clinical KC	The VAE-NT group showed a more pathological CH (8.5 ± 1.5 mmHg) and CRF (8.3 ± 1.5 mmHg) than the normal control group
Atalay et al. [32]	Turkey	27.3 ± 9.5	56	SKC (62, 62)	No slit-lamp findings	Normal topography, Pentacam topometric indices, normal or borderline BAD-D index (< 3.0 SD), back (≤ 16 μm) and front (≤ 7 μm) elevation difference	–	Clinical KC	The final model (AUROC 0.948, sensitivity 87.1%, and specificity 91.4%) chose CH and the BAD-D index The final model had a higher AUROC compared with BAD-D (0.933) and CH (0.80) alone
Galletti et al. [33]	Argentina	34 ± 11	63	SKC	No slit-lamp findings	KSS 0, 1, or 2	–	Clinical KC	Multivariate analysis of ORA signals did not surpass simpler models in SKC detection. Considerable overlap between normal and ectatic eyes irrespective of the analysis model

*FFKC* = forme fruste keratoconus; *SKC* = subclinical keratoconus; *VAE-NT* = very asymmetric ectasia where one eye has clinical keratoconus and the fellow eye appears topographically normal; *KC* = keratoconus; *KISA%* = keratoconus percentage; *CDVA* = corrected distance visual acuity; *CH* = corneal hysteresis; *CRF* = ocular response analyser; *AUROC* = area under the receiver operating characteristic curve; *K* = keratometry; *CCT* = central corneal thickness; *BAD-D* = Belin/Ambrósio enhanced ectasia display total deviation; *ACE-NT* = asymmetric contralateral corneal ectasia with normal topography; *SD* = standard deviation

^*^Keratoconus percentage (KISA%) index: an algorithm that topographically quantifies the phenotypic features of keratoconus [39]; † Keratoconus Severity Score (KSS): a grading system using common clinical markers plus 2 corneal topographic indices, creating a 0 to 5 severity score [40]

Receiver operating characteristic (ROC) curves can be used to determine the predictive accuracy of the test parameters, as described by the area under the curve, and to calculate the sensitivity and specificity of these parameters. Using ROC analysis, Ayar et al. showed that area under the ROC curve (AUROC) values for CH and CRF needed to distinguish FFKC from normal eyes were 0.768 and 0.866, respectively [24]. However, Kirgiz et al. reported slightly higher AUROC values of CH (0.85) and CRF (0.90) to distinguish between the two groups [27]. In a study by Luz et al. investigating the use of different ORA exported signals, waveform parameters, and Pentacam HR metrics to distinguish FFKC from normal corneas, it was found that the Pentacam HR Belin/Ambrósio enhanced ectasia display (BAD), derived from corneal pachymetry, showed the highest predictive value (AUROC, 0.910 ± 0.057), followed by the ORA waveform measurement of the upper 50% area of the first peak (p1area1) (AUROC, 0.717 ± 0.065). However, further analysis revealed that FFKC screening could be significantly enhanced using a combined biomechanical and tomography approach (AUROC, 0.953 ± 0.024), rather than comparing individual parameters [28].

In a study of 61 patients with confirmed definite keratoconus in one eye and asymmetric contralateral corneal ectasia with normal topography (ACE-NT) in the other, it was shown that CRF (0.866) had a higher detection ability (cut-off ≤ 9.6) than CH (0.826) (cut-off ≤ 9.9) for differential diagnosis of normal from ACE-NT eyes [29]. Although most studies have found differences in CH and CRF between early keratoconus and normal eyes, a few studies have found no differences [30]. In VAE-NT eyes, the tomographically normal partner eye showed a significantly more pathological CH (8.5 ± 1.5 mmHg) and CRF (8.3 ± 1.5 mmHg) than the normal control group [31]. Atalay et al. showed that a combined biomechanical and tomographic approach using ORA CH and Pentacam Belin/Ambrósio enhanced ectasia display total deviation (BAD-D) index was optimal for detecting SKC, with the resulting AUROC, sensitivity and specificity (0.948, 87.1%, and 91.4% respectively) being higher than any other individual ORA or Pentacam parameter [32]. The multivariate analysis of ORA signals did not surpass simpler models in early keratoconus detection, and there was considerable overlap between normal and ectatic eyes, irrespective of the analysis model [30, 33].

As outlined above, multiple retrospective and prospective studies [31, 32, 34, 35] have shown that developing a combined biomechanical and tomographic model with multiple variables, may further improve detection of early keratoconus. With increasing complexity of diagnostic technologies, artificial intelligence (AI) has emerged as a promising tool to enhance keratoconus detection and improve accuracy, efficiency, and scalability [36, 37]. However, the development of AI models requires large and diverse data sets, meticulous planning of data inclusion criteria to avoid bias, and detailed database searching, data preprocessing, algorithm selection, testing and validation. A recent review of AI algorithms demonstrated the high degree of accuracy that AI can provide for the detection of manifest keratoconus (~ 98%) [38]. However, the accuracy for detecting subclinical keratoconus was found to be slightly lower (~ 90%), presenting a greater risk of missed diagnosis in patients without clinical signs. As AI systems continue to develop and improve in accuracy for the detection of early keratoconus, it was envisaged that they will become increasingly incorporated into clinical practice.

## Corneal visualization Scheimpflug tonometer (Corvis ST)

The Corvis ST (Oculus, Wetzlar, Germany) is a noncontact tonometer that uses an ultra-high-speed Scheimpflug camera to capture over 4,300 frames per second, allowing the monitoring of corneal response to a metered, collimated air pulse with symmetrical fixed profile and a fixed maximal internal pump pressure of 25 kPa. The Scheimpflug camera has a blue light LED (475 nm, UV free) source and horizontally covers 8.5 mm of a single slit. Recording measurement time is 30 ms [42]. This also allows a more detailed description of the dynamic deformation process in comparison with the ORA system [43].

Corneal biomechanical properties using the Corvis ST can be presented as dynamic corneal response parameters associated with biomechanically adjusted intraocular pressure (bIOP), including applanation length (A1L and A2L), applanation velocity (A1V and A2V) and applanation time (A1T and A2T) at the first and second applanation, the distance between the two peaks, and axial displacement of the apex of the cornea [deformation amplitude (DA) and central radius of the cornea at the highest concavity phase, stiffness parameter at the first applanation (SP-A1), integrated radius (IR), the Ambrósio relational thickness (ART), and deformation amplitude ratio (DA ratio 2 = DA at the apex/average of DA at 2 mm around the center in the horizontal directions]. In addition, the combined biomechanical parameters include the corneal biomechanical index (CBI), tomographic and biomechanical index (TBI), and most recently, the stress-strain index (SSI).

ART is the division between corneal thickness at the thinnest point and the Pachymetric Progression Index. A lower value indicates a thinner cornea and/or a faster thickness increase toward the periphery [44]. SP-A1 uses the displacement between the undeformed apex and the position of first applanation. SP-A1 is defined as the resultant pressure (Pr) divided by deflection amplitude at A1. Resultant pressure (Pr) is defined as the adjusted pressure at A1 (adj AP1) minus a biomechanically adjusted IOP value (bIOP), resulting in the following equation: SP-A1 = (adjusted AP1–bIOP)/A1 deflection amplitude. The spatial and temporal profiles of the Corvis ST air pulse are measured using hot wire anemometry [44, 45]. CBI is a logistic regression algorithm that combines different biomechanical parameters to optimize the separation keratoconus eyes and normal eyes. It includes several dynamic corneal response parameters: deformation amplitude ratio (DA ratio) at 1 and 2 mm, applanation 1 velocity, the standard deviation of deformation amplitude at highest concavity, Ambrósio relational thickness to the horizontal profile (ARTh), SP-A1, and IR. With a cutoff value of 0.5 using CBI, 98.2% of the cases were correctly classified with 100.0% specificity and 94.1% sensitivity in differentiating definite keratoconic patients from the normal population [44]. TBI is calculated using an AI approach to optimize ectasia detection. By combining tomographic data from the Pentacam HR with biomechanical data from the Corvis ST, one can further improve sensitivity and specificity in the detection of patients with a significant risk for developing ectasia after refractive surgery [35]. The SSI was generated based on predictions of corneal behaviour using numerical finite element modelling, which simulates the effects of IOP and the Corvis ST air puff to estimate the stiffness of the material [46]. The newly developed SSI II (SSI map) provides an estimation of the regional variation of biomechanical stiffness across the corneal surface and it is anticipated that these maps could be particularly useful in understanding keratoconus development and progression [47].

### Corvis ST in FFKC

Although some clinicians may misuse the term of ‘topography’ and ‘tomography’, the most widely used pre-surgery screening technique of detecting corneal topographic/tomographic information is relying on both the anterior and posterior surface, by Scheimpflug based and Placido-disk based instruments. Hence, in FFKC, using these modalities, the topographic/tomographic indices look the same as normal eyes, and the only distinctive parameter between them is the abnormal ectatic contralateral eye [29]. Therefore, the biomechanical parameters might be helpful in such cases.

Hwang et al. [48] reported that the maximum ART (ARTmax) yielded an AUROC of 0.739 (sensitivity of 56.7% and specificity of 88.3%), to distinguish the less affected eye of VAE (the same definition as VAE-NT) from normal eyes. Awad et al. [49] also found that ART (AUROC: 0.88) was a highly sensitive objective parameter in FFKC cases. However, Shajari et al. [50] demonstrated ARTmax (AUROC: 0.613) performed less well in differentiating populations at early stages of keratoconus. They proposed that using index of height decentration and index of vertical asymmetry were better markers in the early stages of keratoconus, and when the disease progresses, the BAD-D index is better suited to diagnose ectasia [50].

Some researchers have found that corneal biomechanical properties are highly sensitive for the detection of early keratoconus even in the absence of topographic abnormalities. After controlling for CCT and bIOP, the values of corneal deflection amplitude during the first applanation, A1L, corneal deflection amplitude during the second applanation, and maximum deformation amplitude increased in the FFKC compared with the normally thin cornea [51]. Tian et al. also found that A1T was faster, and the SP-A1 and CBI were significantly softer in FFKC cases compared to the corneal thickness and IOP in age-matched healthy corneas [52]. Among the top five indices of the AUROCs for detecting early keratoconus (similarly to FFKC), the corneal biomechanical-related index accounted for 80%, including the A1 dArc length (AUROC: 0.901), highest concavity radius (AUROC: 0.879), A2T (AUROC: 0.877), and TBI (AUROC: 0.874) [53]. The BAD-D also provided a high predictive value (AUROC: 0.91 ± 0.057) [28]. Another study in a Chinese population confirmed the discriminatory values of TBI (AUROC: 0.928, cutoff: 0.38, Youden index: 0.753), and CBI (AUROC: 0.860, cutoff: 0.27, Youden index: 0.642) for distinguishing FFKC from normal eyes [54]. Significant differences were found for A2L, A1V, A2V, and TBI between subclinical keratoconus (similarly to FFKC) and normal eyes. TBI showed the highest AUROC (0.790; cutoff: 0.29; sensitivity: 67%; specificity: 86%) in distinguishing subclinical keratoconus from normal eyes [55].

Although corneal biomechanical stability was found to be significantly lower in FFKC eyes than in normal eyes, with significantly decreased SP-A1 and increased TBI, the AUROCs of SP-A1 and TBI for identifying FFKC were lower than 0.7 [56]. Wang et al. found that comparable AUROC and partial AUROC was observed between the CBI (AUROC: 0.785; pAUROC: 0.079) and BAD-D (AUROC: 0.757; pAUROC: 0.068) for detecting FFKC with sensitivities of 63.2% and 52.6%, given a common specificity of 80.3%, which is lower than in the advanced keratoconus cases [57]. Tian et al. established the keratoconus diagnosis model using backpropagation neural network, and found that the predicted value (AUROC: 0.877) was more sensitive in the detection of FFKC than CBI (AUROC: 0.610) and TBI (AUROC: 0.659) [52]. Luz et al. [28] also found that the logistic regression model yielded the highest accuracy (AUROC: 0.953, sensitivity: 85.71%, specificity: 98.68%). This model incorporated variables such as BAD-D, ARTmax, and the thinnest point-related elevation on both the anterior and posterior corneal surfaces [28].

In a study involving 137 patients, SSI was found to differ significantly between keratoconus, FFKC and normal eyes, indicating an independent decrease in corneal stiffness in eyes with keratoconus. However, the AUROC and Youden index of the SSI were not as good as TBI (AUROC: 0.928), BAD-D (AUROC: 0.926) and CBI (AUROC: 0.860) in detecting FFKC [54]. After correcting for CCT and bIOP, SSI II and ART were significantly higher, and CBI was significantly lower in the normal group than in the FFKC group, SKC group and clinical keratoconus groups. AUROC of SSI II was significantly higher than all other Corvis parameters in distinguishing normal eyes from FFKC, followed by ART and CBI [58].

In a secondary statistical analysis of 80 publications utilizing Pentacam or Corvis ST parameters, it was found that except for CBI, SPA1 was the only Corvis ST output parameter sensitive to FFKC (AUROC: 0.87, sensitivity: 0.71, and specificity: 0.85). SP-A1 was not inferior to the CBI and may be the earliest Corvis ST output to reflect changes in corneal biomechanics during keratoconus progression. Furthermore, it was found that most of the 20 sensitive diagnostic parameters screened that were related to the thinnest point of the cornea, such as thickness progression parameters, height parameters based on the thinnest point, and even thinnest point of the cornea itself, were selected as the sensitive diagnostic parameters of FFKC [59].

### Covis ST in subclinical keratoconus

Although clinically, both SKC and FFKC are not evident on slit-lamp examination, the biomechanical parameters A1T, IR, and TBI are significantly varied between the SKC and FFKC, suggesting that these parameters are more powerful for detecting subtle changes in corneal biomechanical properties than SP-A1. Overall, the biomechanical properties of SKC are weaker than FFKC [60].

In an age controlled normal vs. SKC study, Peris-Martinez et al. evidenced the usefulness of the biomechanical parameters provided by Corvis ST in detecting subclinical keratoconus, showing statistically significant differences in maximum deformation amplitude, highest concavity radius, A2L and A2V [61]. In addition, others found that A1L, A2L, radius of the inward-bended cornea, and deflection length at the highest concavity parameters demonstrated statistically significant differences [62]. Table 2 provides a summary of studies examining early keratoconus detection with Corvis ST.

**Table 2:** Previous studies on early keratoconus with Corvis ST

Authors	Country	Age (years)	Male (%)	Cases, eyes	Definition of FFKC				Main results
					Slit-lamp examination	Topography	Others	Fellow eye	
Hwang et al. [48]	US	31.8 ± 13.4	56.6	VAE-NT (AKC in the original text) (30, 30)	No findings	No	CDVA of 20/20	Clinical KC	ART max yielded an AUROC of 0.739 (sensitivity of 56.7% and specificity of 88.3%)
Awad et al. [49]	Egypt	30.6 ± 9.2 (14–44)	–	FFKC (48, 48)	Normal	Normal topography (Pentacam), mean K < 47 D, I-S ≤ 1.4 D	–	Clinical KC	ART (AUROC 0.88) was a highly sensitive parameter in the FFKC cases
Shajari et al. [50]	Germany	Matched control group (32 ± 11)	–	FFKC (normal tomography in the original text) (27, 27)	Normal	Normal topography (according to elevation maps, corneal thickness, maximum K, and D-index)	–	Clinical KC	ARTmax (AUROC 0.613) was not strong in differentiating populations at early stages of keratoconus
Zhang et al. [51]	China	21 ± 9	43.5	FFKC (23, 23)	Normal	Normal topography with no asymmetric bowtie and no focal or inferior steepening pattern	–	Clinical KC	Corneal deflection amplitude during the first applanation, length at the first applanation, corneal deflection amplitude during the second applanation, and maximum deformation amplitude $\uparrow$ in FFKC
Tian et al. [52]	China	23.6 ± 8.7	–	FFKC (36, 36)	Normal	No abnormal or suspect tomography	–	Clinical KC	A1T, SP-A1, CBI were significantly softer in the FFKC; diagnosis model using backpropagation neural network (AUROC 0.877) was more sensitive in the detection FFKC than the CBI (AUROC 0.610) and TBI (AUROC 0.659)
Chen et al. [53]	China	22.93 ± 4.91 (12–34)	70.3	FFKC (91, 91)	Normal	Corneal tomography was relatively normal (no asymmetrical bowtie type—oblique radial axis and no central or lower area steep), I-S < 1.4 D, KISA% < 60%)	–	Clinical KC	A1 dArc length (AUROC 0.901), highest concavity radius (AUROC 0.879), A2T (AUROC 0.877), and TBI (AUROC 0.874)
Luz et al. [28]	Brazil	25.5 ± 7.2	–	FFKC (21, 21)	Normal	KISA% < 60% (Placido-disk topography) without a suspect pattern	–	Clinical KC	BAD-D alone (AUROC 0.91 ± 0.057), highest AUROC (AUROC 0.953, sensitivity 85.71%, specificity 98.68%) for a logistic regression model by adding BAD-D, ART Max, and thinnest point related elevation on both the front and back surface
Liu et al. [54]	China	22.00 ± 6.26	59.3	VAE (27, 27)	Normal	Normal topography (mean K < 47 D and I-S value ≤ 1.4 D)	CDVA of 20/20, thickness at thinnest point > 470 μm	Clinical KC	The AUROC of the SSI were not as good as TBI (AUROC 0.928), BAD-D (AUROC 0.926) and CBI (AUROC 0.860)
Koc et al. [55]	Turkey	27.7 ± 6.9	47.6	SKC (21, 21)	Normal	Normal topographic, normal topometric and tomographic findings	–	Clinical KC	TBI AUROC (0.790, cut-off 0.29, sensitivity 67%, specificity 86%). Significant differences were found in the values of A2L, A1V, A2V, and TBI
Xian et al. [56]	China	–	–	FFKC (44, 44)	Normal	Topographic (paracentral I-S ≤ 1.4 D), and tomographic (central anterior and posterior elevations < 8 μm and 13 μm, respectively) examinations	With the Best-Fit-Sphere as the reference sphere	Clinical KC	↓ SP-A1 and $\uparrow$ TBI in FFKC eyes but AUROCs of SP-A1 and TBI were lower than 0.7
Wang et al. [57]	US	33.8 ± 10.6	–	FFKC (21, 21)	Normal	No tomographical signs	–	Clinical KC	CBI (AUROC 0.785, sensitivity 63.2%) and BAD-D (AUROC 0.757, sensitivity 52.6%) with a common specificity of 80.3%
Miao et al. [58]	China	22.76 ± 4.99	75.3	FFKC (194, 194)	Normal	Normal topography (mean K < 47.00 D; KISA% < 60%; I-S < 1.4 D)	–	Clinical KC	AUROC of SSI II was the highest in distinguishing normal eyes from FFKC, followed by ART and CBI
Zhang et al. [59]	Secondary analysis	–	–	–	–	–	–	–	SP-A1 (AUROC 0.87, sensitivity 0.71, and specificity 0.85) was the only Corvis ST output parameter sensitive to FFKC except the CBI
Peris-Martinez et al. [61]	Spain	Men (26 ± 13) Women (31 ± 19)	61.5	SKC (13, 16)	Normal	Topography normal with no asymmetric bowtie, and no focal or inferior steepening pattern	–	Clinical KC	Significant differences were found in A1T and A2T, maximum deformation amplitude, highest concavity radius, and A2L and A2V
Steinberg et al. [62]	Germany	31 ± 10	–	SKC (NA, 27)	Normal	KISA% index < 60%, I-S < 1.4 D, and Kmax ≤ 47 D	–	Clinical KC	None of the A1L, A2L, radius of the inward-bended cornea, and deflection length at the highest concavity parameters showed differences
Castro-Luna et al. [63]	Spain	40.21 ± 13.19	–	SKC (20, 20)	No slit-lamp findings	Minor topographic signs of keratoconus and suspicious topographic findings (mild asymmetric bowtie, with or without deviation)	Mean K < 46.5 D; minimum corneal thickness > 490 μm;	Clinical KC	SP-A1 and A2T were the most critical determinants. The random forest model was a good model for classifying SKC (specificity 93%, sensitivity 86%)
Ren et al. [64]	China	22.79 ± 5.78	–	SKC (100, 100)	Normal	No clear evidence of KC	–	Clinical KC	SP-A1 (AUROC 0.753) and CBI (AUROC 0.703) showed significant differences between normal and SKC eyes
Heidari et al. [65]	Iran	30.15 ± 5.42	–	SKC (79, 79)	No finding	Abnormal findings in topography and tomography maps	–	Clinical KC	AUROCs for SP-A1 (0.779), ARTh (AUROC 0.718, CBI (AUROC 0.758), and TBI (AUROC 0.828), were all inferior to the Sirius symmetry index of back (0.908) and Pentacam I-S value (0.862)
Chan et al. [66]	China	–	–	SKC (23, 23)	Normal appearing	Atypical or suspect topography findings that did not meet the diagnostic criteria for KC, with average K ≤ 49 D or HOAs ≤ 1.50 µm in either eye or normal topography	–	Clinical KC	Significant differences were found in BAD-D and TBI between SKC and normal. The TBI and BAD-D showed the highest AUROC (0.925 and 0.786)
Song et al. [67]	China	24.87 ± 7.36	52.9	SKC (70, 70)	Normal	I-S asymmetry and/or bowtie pattern with skewed radial axes (with/without)	–	Clinical KC	AUROC of TBI and BAD-D was 0.944 and 0.965, respectively
Augustin et al. [69]	Germany	27.4 ± 9.3	100	VAE-NT (14, 14)	Normal	Tomographically normal eyes (BAD-D < 1.6 and regular ABCD grading system*)	–	Clinical KC	High sensitivity of both CBI (99.1%) and TBI (99.6%) in detecting tomographic abnormal keratoconus
Herber et al. [70]	Italy	–	–	VAE-NT (18, 18), VAE-NTT (55, 55)	Normal	Topographically normal (VAE-NT), topographically and tomographically normal (VAE-NTT)	–	Clinical KC	CBI provided high sensitivity and specificity of 93.1% to distinguish normal eyes from VAE-NT and VAE-NTT using a cut-off value of 0.2
Ambrósio et al. [35]	Brazil, Italy	–	57.4	VAE-NT (94, 94)	Normal	KISA% < 60%, I-S < 1.45 D	–	Clinical KC	The AUROCs for the TBI, BAD-D, and CBI were 0.985, 0.839, and 0.822 in the VAE-NT group. A TBI cut-off value of 0.29 provided 90.4% sensitivity with 96% specificity
Kataria et al. [71]	India	22 ± 10	–	VAE-NT (also written as SKC) (100, 100)	Normal	VAE with normal topography (TMS-4), KISA% < 60%, I-S < 1.45 D	–	Clinical KC	The TBI (AUROC 0.90) was superior to CBI (AUROC 0.78), BAD-D (AUROC 0.81) and SP-A1 (AUROC 0.76). The TBI (with a 0.63 cutoff) showed the highest accuracy (99.5%), with 99% sensitivity, 100% specificity
Wallace et al. [72]	New Zealand	26.2 ± 10.1	67	VAE-NT (21, 21)	Normal	VAE with normal tomography (Pentacam), KISA% < 60%, I-S < 1.45 D	–	Clinical KC	The TBI (AUROC 0.92) was superior to CBI (AUROC 0.78) and BAD-D (AUROC 0.81). At a cutoff of 0.72, TBI has 99% sensitivity, 67% specificity, and 92% accuracy
Salomao et al. [73]	Brazil, Italy	–	–	VAE-NT (NA, 125)	Normal	VAE with relatively normal topography	–	Clinical KC	AUROC of the TBI was 0.966, BAD-D (0.834), CBI (0.774)
Sedaghat et al. [29]	Iran	26.2 ± 4.3	42	ACE-NT (128, 128)	Normal	Normal pattern and index (Pentacam)	–	Clinical KC	TBI has the best accuracy (AUROC 0.966) for differential diagnosis with a cutoff of 0.24
Steinberg et al. [34]	Germany	–	–	VAE-NT (NA, 32) VAE-NTT (NA, 18)	Normal	VAE with regular topography (VAE-NT) or regular topography and tomography (VAE-NTT)	–	Clinical KC	The accuracy was reproducible (accuracy in current study population with an optimized TBI cut-off: 0.72), to differentiate between normal and VAE-NT in the study population
Ferreira et al. [74]	Brazil	33.26 ± 14.41	59.6	VAE-NT (57, 57)	Normal	Normal topography (KISA% < 60% and I-S < 1.45 D)	–	Clinical KC	In the VAE-NT group, optimized TBI cut-off value of 0.295 provided a sensitivity of 89.5% and a specificity of 91.0% (AUC 0.960; 95% CI 0.937–0.983)
Koh et al. [75]	Japan	47.2 ± 10.2	–	VAE-NT (23, 23)	Normal	Normal topography (TMS-4)	–	Clinical KC	The AUROC for the BAD-D, CBI, and TBI were 0.668, 0.660, and 0.751, respectively. The TBI cut-off of 0.259 provided 52.17% sensitivity and 88.57% specificity. Nine VAE-NT cases (39.1%) exhibited normal values for the BAD-D, CBI, and TBI. 40% of VAE-NT eyes were classified as normal by the BAD-D, CBI, and TBI
Fraenkel et al. [31]	Germany	33.6 ± 13.3	65	VAE-NT (26, 26)	Normal	Tomographically normal part eye in very asymmetrical corneal ectasia	–	Clinical KC	TBI of the VAE-NT (0.19 ± 0.25) did not differ significantly. Five (19.2%) of 26 eyes had a TBI more than 0.29 and were considered pathological. The VAE-NT eyes (8.5 ± 1.5 mm Hg) showed a significantly more pathological CH and CRF (8.3 ± 1.5 mm Hg) compared with the normal eyes
Augustin et al. [76]	Germany	–	–	VAE-NT (34, 34)	Normal	Tomographically regular fellow eyes by Pentacam AXL	–	Clinical KC	The TBI showed slightly higher sensitivity than the CBI (62% vs. 53%) for detecting keratoconus. 21% of the keratoconus partner eyes could not be recognized as conspicuous, either by CBI or TBI
Padmanabhan et al. [77]	Italy, Brazil	34.29 ± 14.28	–	VAE-NT 105, 105)	Normal	Fellow eye with normal topography (Pentacam HR)	–	Clinical KC	No differences in the SSI were observed between healthy individuals and VAE-NT cases

*FFKC* = forme fruste keratoconus; *VAE-NT* = very asymmetric ectasia where one eye has clinical keratoconus and the fellow eye appears topographically normal; *AKC* = asymmetric keratoconus; *CDVA* = corrected distance visual acuity; *KC* = keratoconus; *SKC* = subclinical keratoconus; *ART* = Ambrósio relational thickness; *AUROC* = area under the receiver operating characteristic curve; *K* = keratometry; *I-S* = inferior-superior asymmetry value; *A1T* = time to reach the first applanation; *SP-A1* = stiffness parameter at the first applanation; *CBI* = corneal biomechanical index; *TBI* = tomographic and biomechanical index; *KISA%* = keratoconus percentage; *A1 dArc* = A1 delta Arc length; *A2T* = time to reach the second applanation; *BAD-D* = Belin/Ambrósio enhanced ectasia display total deviation; *VAE* = very asymmetric ectasia; *SSI* = stress–strain index; *A2L* = length at the second applanation; *A1V* = velocity at the first applanation; *A2V* = velocity at the second applanation; *ARTh* = Ambrósio relational thickness to the horizontal profile; *HOAs* = high-order aberrations; *VAE-NTT* = very asymmetric ectasia where one eye has clinical keratoconus and the fellow eye appears topographically and tomographically normal; *ACE-NT* = asymmetric contralateral corneal ectasia with normal topography; *CH* = corneal hysteresis; *CRF* = corneal resistance factor

^*^ABCD keratoconus grading system [89]

Random forest machine learning techniques could advance the classification and prediction of SKC through developing an algorithm for mining the metrics generated by the Pentacam HR and Corvis ST. The random forest approach is deemed a good model for classifying SKC, demonstrating a specificity of 93% and sensitivity of 86% [63]. SP-A1 can become a critical determinant in classifying and identifying SKC when compared to maximum deformation amplitude radius at 2 mm and 1 mm, IR, ARTh and CBI, followed by A2T [63]. In line with this, SP-A1 and CBI were significantly different between normal control and SKC eyes, and the parameter with the highest diagnostic efficiency was SP-A1 (Youden index: 0.40, AUROC: 0.753), followed by CBI (Youden index: 0.38, AUROC: 0.703), and IR (Youden index: 0.33, AUROC: 0.668) [64]. Heidari et al. [65] reported close AUROCs for SP-A1 (0.779), and lower AUROCs for ART (0.718), CBI (0.758), and TBI (0.828), which were all inferior to the Sirius symmetry index of back (0.908) and Pentacam HR I-S value (0.862) in differentiating SKC from normal eyes.

A significant difference was also found in the BAD-D and TBI between normal and SKC eyes [66]. However, whilst some found TBI to be optimal for the detection of SKC (AUROC: 0.925 for TBI vs. 0.786 for BAD-D) [66], others found that BAD-D provided a slightly better diagnostic performance (AUROC: 0.944 for TBI vs. 0.965 for BAD-D) [67]. Interestingly, AI-driven approaches, when integrated with advanced tools like the Scheimpflug rotation camera-based elevation map, Placido-disk-based keratometry, and extensive databases, show great promise in identifying early-stage keratoconus. However, the role of clinical experts remains irreplaceable, especially when making critical decisions prior to refractive surgery. That said, there may still be some concerns regarding the level of experience among clinical specialists in leveraging these technologies effectively [68].

### Corvis ST in VAE-NT eyes

Objective criteria for considering normal topography was rigorously applied for defining cases of VAE-NT, and included objective front surface curvature metrics derived from the Pentacam HR, such as a keratoconus percentage index (KISA%) score lower than 60% and a paracentral inferior-superior (I-S value) asymmetry value at 6 mm (3-mm radii) less than 1.45 D [35, 39].

In a recent study, in which 14 patients with VAE-NT were enrolled (although the tomographically normal fellow eyes of keratoconus patients are rare), regular CBI values (0–0.249) were found in 6/14 of the VAE-NT eyes examined. The mean TBI was 0.47 ± 0.22 (range: 0.22–0.84) with regular TBI values (0–0.249) recorded in only 2/14 patients. The sensitivity of both, CBI (99.1%) and TBI (99.6%) in detecting tomographic abnormal keratoconus can be considered very high [69]. Furthermore, another study comparing the use of biomechanical indices generated by the ORA and Corvis ST (a dynamic Scheimpflug analyser) to distinguish between normal eyes and those with very asymmetric ectasia [defined as unilateral keratoconus, where one eye has clinical keratoconus and the fellow eye appears topographically normal (VAE-NT) or topographically and tomographically normal (VAE-NTT)], it was found that the Corvis biomechanical index had the highest AUROC (0.979), followed by ORA CRF (0.865) and CH (0.824) [70]. The CBI (cutoff 0.2) showed a sensitivity of 100.0% and 70.9%, respectively, and a specificity of 93.1% for distinguishing normal eyes from VAE-NT and VAE-NTT, whereas the values for ORA CRF and CH were much lower [70].

TBI combines Scheimpflug-based corneal tomography and biomechanics for enhancing ectasia detection. A retrospective analysis of Corvis HR data from the eyes of 684 patients revealed the AUROCs for TBI, BAD-D, and CBI to be 0.985, 0.839, and 0.822 in discriminating VAE-NT eyes from normal controls [35]. This superior performance of TBI over CBI and BAD-D in distinguishing VAE-NT from normal corneas is also supported by several groups [71–73]. Although high accuracy (> 85%) has been reported in many studies, a number of cutoff values exist ranging from 0.16 [66], 0.24 [29], 0.29 [35], 0.63 [71], 0.72 [34, 72], 0.295 [74], to 0.259 [75].

Fraenkel et al. examined ORA and Corvis ST parameters in normal and VAE-NT eyes and provided evidence of pathological CH (8.5 ± 1.5 mm Hg) and CRF (8.3 ± 1.5 mm Hg) in the VAE-NT eyes. Although the average TBI value for the VAE-NT eyes (0.19 ± 0.25) did not differ significantly from the normal eyes, 5/26 VAE-NT eyes (19.2%) had a TBI of more than 0.29 and were considered pathological [31]. In larger study by Sedaghat et al. it was shown that CRF (AUROC: 0.866) had a higher detection ability than CH (AUROC: 0.826) in distinguishing normal eyes from VAE-NT eyes, and the discriminative analysis showed that the highest accuracy of the Corvis ST was related to TBI (0.966) [29].

However, controversial results were observed for differentiating normal and VAE-NT eyes. The AUROC for the BAD-D, CBI, and TBI were 0.668, 0.660, and 0.751, respectively. The TBI cut-off of 0.259 provided a sensitivity of 52.17% and a specificity of 88.57% [75]. Nine VAE-NT cases (39.1%) were found to exhibit normal values for BAD-D, CBI, and TBI. Moreover, 40% of VAE-NT eyes were classified as normal through the use of BAD-D, CBI, and TBI [75]. Although some of these cases may truly represent unilateral ectasia, further advances are needed to enhance ectasia detection and characterize the susceptibility for ectasia progression. In another observational study, of more than 900 participants, 34 eyes with VAE-NT (7.4%) were identified and included in the analysis. Biomechanical analysis demonstrated a mean CBI of 0.28 ± 0.26 and a mean TBI of 0.34 ± 0.30. Out of the 34 eyes, 16 (47%) normal CBI values, 13 (38%) regular TBI and 7 (21%) regular TBI and CBI were observed. The sensitivity of CBI and TBI in detecting a tomographically normal keratoconus fellow eye was 53% and 62%, respectively. TBI showed slightly higher sensitivity than the CBI (62% vs. 53%) for detecting keratoconus in a tomographically unremarkable keratoconus partner eye. Further, 21% of the keratoconus partner eyes could not be recognized as conspicuous, either by CBI or TBI [76]. Even though the SSI value decreased with keratoconus progression, no differences in SSI were observed between healthy individuals and VAE-NT cases, which may be due to the focal nature of keratoconus [77].

The index of height asymmetry and height decentration differed significantly between FFKC and mild keratoconus eyes and thin normal corneas. The index of height decentration also had sufficient strength (AUROC > 0.80) to differentiate FFKC and mild keratoconus from thin normal corneas eyes. The deflection amplitude of the first applanation showed a good potential to differentiate (AUROC > 0.70) FFKC and mild keratoconus from thin normal corneas [78]. Central astigmatism from the anterior corneal surface had an AUROC of 0.862 for distinguishing FFKC from normal eyes. This was associated with a cutoff point of 4.65, providing 73.5% sensitivity and 99.3% specificity. Conversely, other combined parameters, including CBI, TBI, and BAD-D, had low AUROCs to differentiating FFKC from normal eyes [79].

Zhang et al. [80] applied a stepwise logistic regression model to differentiate FFKC from normal eyes. The model was derived from the parameter ‘elevation of front surface in thinnest location’ from the Pentacam and deflection amplitude of the highest concavity and SP-A1 from the Corvis ST. The predictive accuracy of this model presented an excellent AUROC (0.965) with 100.0% sensitivity and 84.0% specificity, followed by TBI (0.885), elevation of front surface in thinnest location (0.874) and BAD-D (0.839) alone. However, the CRF and CH output by ORA did not improve the combined diagnosis, despite the corneal combination of morphological and biomechanical properties that optimized the diagnosis of FFKC [80].

In summary, the diverse screening methods used across various studies mean that participants in each study may reflect different stages of ectatic disease. The Corvis ST-derived parameters—whether basic, advanced, or integrated—exhibited varying performance, leading to a wide spectrum of cutoff values for each index.

### Development based on the current Corvis ST indices

A notable limitation of the Corvis ST in the screening of keratoconus is that it automatically images a single, central 8.5 mm horizontal section of the cornea [55, 81]. Given that a common location of focal thinning in keratoconus is the inferotemporal site, the Corvis ST may therefore fail to capture the focal area of corneal biomechanical compromise in keratoconus [82, 83]. Kenia et al. found that in a subclinical keratoconus group, combining epithelial mapping with corneal biomechanical parameters could help improve the efficacy in the diagnosis of SKC [84]. Furthermore, a retrospective comparative study of 33 clinically unaffected eyes characterised by normal corneal topography and biomechanical properties, revealed abnormal pachymetric patterns using Pentacam within the central cornea, alongside subtle morphological alterations evident in early-stage keratoconus with preserved biomechanics [85]. These findings collectively underscore the potential of morphological parameters, specifically those related to epithelial characteristics and pachymetric distribution, to furnish supplementary diagnostic insights into the early stages of keratoconus.

Additionally, due to the distribution range of many biomechanical parameters overlapping between normal individuals and SKC patients, it was hypothesised that introducing biomechanical interocular asymmetry assessment may reduce the false-negative rate and improve the sensitivity of diagnosing SKC [86]. Indeed, this was demonstrated using the binocular assisted biomechanical index, which produced an AUROC of 0.998 (sensitivity: 97.8%, specificity: 99.2%; cutoff: 0.401), which was statistically higher than CBI (AUROC: 0.935, sensitivity: 85.6%) [86].

Finally, significant differences in the values of SP-A1 and SSI and ART were noticed between the Caucasians and the Chinese [87]. A relatively high false rate for keratoconus suspects was also found in the Chinese population [54]. The new CBI optimized for Chinese patients (cCBI) was investigated and showed an AUROC of 0.990. With a cutoff value of 0.5, it produced 93.7% specificity and 95.9% sensitivity in the training dataset for distinguishing healthy eyes from typical keratoconus eyes. Conversely, the original CBI produced an AUROC of 0.981 with 75.4% specificity and 97.9% sensitivity [88]. It is important to note that in this new cCBI study, they excluded unilateral keratoconus, FFKC, and SKC from the databases when creating and validating the cCBI. More studies are warranted to test the capability of cCBI to separate healthy individuals from those with FFKC and other early stages of keratoconus in Chinese patients.

## Optical coherence tomography (OCT)

Anterior segment OCT is another noncontact high-resolution imaging technique available for three-dimensional anterior segment imaging in keratoconus [90]. Since its introduction in the 1990’s, significant advances have been made in OCT imaging of the cornea. Time-domain OCT was initially developed for capturing cross-sectional images of the eye’s anterior segment [91]. Subsequently, 840 nm spectral-domain OCT was introduced, with a faster image acquisition and better resolution; however, this advancement led to more limited image depth ranges and horizontal scan widths. Additionally, other variations of spectral-domain OCT with improved axial resolutions, termed ultra-high-resolution OCT have been developed. Currently, a 1310 nm swept-source OCT based on the Fourier-domain OCT type allows for more precise and quicker reconstruction of three-dimensional anterior segment images [90]. A summary of currently commercially available OCT devices is shown in Table 3.

**Table 3:** Summary of currently commercially available anterior segment optical coherence tomography (OCT)

Type	Instrument	Company	Approximate axial resolution (µm)	Scanning speed (A scans per second)
Time-domain	Stratus OCT	Carl Zeiss Meditec	10	400
	Visante OCT	Carl Zeiss Meditec	18	2000
	Slit-lamp OCT	Heidelberg	25	200
Spectral-domain	Spectralis OCT	Heidelberg	7	40000
	Cirrus HD-OCT	Carl Zeiss Meditec	5	27000
	OCT SLO	Optos	6	27000
	3D OCT-1 Maestro 2	Topcon	6	50000
	3D OCT-2000	Topcon	6	27000
	RTVue-100	Optovue	5	26000
	RTVue XR Avanti	Optovue	5	70000
	iVue-100 and iFusion 80	Optovue	5	80000
	RS-3000 Advance2	Nidek	7	85000
	REVO	Optopol	5	130000
	Envisu C-2300	Leica	3	32000
	MS-39	CSO	3.6	30000
Swept-source	CASIA SS-1000	Tomey	10	30000
	CASIA2	Tomey	30	50000
	DRI OCT Triton	Topcon	8	100000
	ANTERION	Heidelberg	10	50000

### OCT in the early diagnosis with corneal morphology analysis

At present, OCT excels in the identification and digitalization of both corneal surfaces with good repeatability and high reliability [94], providing curvature and elevation maps of the anterior and posterior cornea and corneal thickness (along with corneal pachymetry maps) [95]. Additionally, the advantages of OCT lie in providing clearer images of corneas with opacities using longer wavelength scanning beams [96–98]. Further, the Fourier analysis of corneal power with OCT can quantitatively evaluate corneal irregular astigmatism in the various stages of keratoconus, including advanced keratoconus cases with corneal opacities [99]. Therefore, corneal analysis using OCT can quantify the refractive component, corneal thickness, and irregular astigmatism of the cornea across different stages (very mild to advanced) and may also be used for longitudinal observation.

Parameters for early detection in FFKC, have been studied for both anterior and posterior corneal surfaces with OCT. In previous studies, variables used to define FFKC were reported as keratoconus in the fellow eye in 77.27%, normal topography in 59.09%, and normal slit-lamp examination in 40.90%. Among these studies, 90.90% used more than one parameter to define FFKC. However, topographic signs and parameters used to define FFKC differ between groups, making it difficult to unify them, and will be noted in Table 4 [16].

**Table 4:** Previous studies on forme fruste keratoconus (FFKC) with optical coherence tomography (OCT)

Utilized OCT	Authors	Country	Age (years)	Male (%)	Cases, eyes	Definition of FFKC				Main results
						Slit-lamp examination	Topography	Others	Fellow eye	
RTVue	Pavlatos et al. [100]	US	–	–	26, 26	No signs of KC	Normal topography from Scheimpflug or a scanning slit topographer	CDVA of 20/20 or better	Clinical or subclinical KC	Classification accuracy with ectasia index, computed from pachymetry and posterior surface mean curvature, was 63% ± 21%
	Hwang et al. [48]	US	31.8 ± 13.4	57	30, 30	No signs of KC	No definitive abnormalities with Scheimpflug imaging and OCT	CDVA of 20/20 or better	Clinical KC	No individual OCT metric yielded an AUROC > 0.75. Combining 11 OCT thickness metrics ↑ AUROC to 0.96. Combining Scheimpflug/ OCT metrics ↑ AUROC to 1.0
ANTERION	Saad et al. [95]	France	33.2 ± 7.6	–	43, 43	No signs of KC	No abnormality with specular corneal topography assisted by Nidek Corneal Navigator analysis	CDVA of 20/20 or better	Clinical KC	Among curvature and thickness parameters, I-S value yielded the highest AUROC of 0.850, followed by the magnitude of inferior decentration of posterior steepest keratometry
CASIA SS-1000/ CASIA2	Fukuda et al. [101]	Japan	33.9 ± 14.4	76	25, 25	No signs of KC	No topographic signs of KC	Anterior corneal surface curvature metrics of 0% KCI and 0% KSI through the KC screening program involving Placido-disk corneal topography	Clinical KC	Among anterior and posterior keratometric parameters, elevation, topographic parameters, regular and irregular astigmatism and pachymetric parameters, posterior corneal elevation had the highest predictive accuracy (AUROC of 0.912)
	Kitazawa et al. [103]	Japan	29.5	79	14, 14	No signs of KC	Normal anterior topography map by Scheimpflug imaging	–	Unilateral KC	The anterior–posterior ratio of corneal surface area derived from elevation maps yielded AUROC of 0.986
	Itoi et al. [102]	Japan	28.2 ± 6.4	85	13, 13	No signs of KC	No topographic signs of KC	TMS-4 keratoconus screening program with 0% KCI	Clinical KC	The anterior–posterior ratio of corneal surface area derived from elevation maps showed AUROC of 0.980
	Maeno et al. [99]	Japan	40.7 ± 9.5	68	50, 50	No signs of KC	No topographic signs of KC	Anterior corneal surface curvature metrics of 0% KCI and 0% KSI with the KC screening program involving Placido-disk corneal topography, and the criterion of an I-S value of less than 1.4 D at 6 mm on the topographic map	Clinical KC	In Fourier analysis of corneal power distribution, posterior asymmetry (AUROC 0.778) and higher-order irregularity (AUROC 0.709) showed the best discrimination among single components
	Shiga et al. [105]	Japan	22.0 ± 8.5	78	23, 23	No signs of KC	No topographic signs of KC	Normal corneal tomography, 0% KCI with Placido-disk corneal topography and 0% diagnosis probability with the ectasia screening score obtained by using AS-OCT	Clinical KC	Combining the Fourier posterior corneal asymmetry and central corneal thickness gave an AUROC of 0.893. Adding SP-A1 from biomechanical assessment $\uparrow$ AUROC to 0.947, though there was no significant difference between using each device alone or in combination

*KC* = keratoconus; *CDVA* = corrected distance visual acuity; *AUROC* = area under the receiver operating characteristic curve; *I-S value* = inferior-superior asymmetry value; *KCI* = Klyce/Maeda keratoconus index; *KSI* = Smolek/Klyce keratoconus severity index; *AS-OCT* = anterior segment optical coherence tomography; *SP-A1* = stiffness parameter at the first applanation

For RTVue (a Fourier-domain system from Optovue Inc., Fremont, CA, USA), other than epithelial thickness, ectasia index, computed from pachymetry and posterior surface mean curvature, has been studied by Pavlatos et al. in FFKC [100]. The results have shown that the subtle early signs of keratoconus may vary between cases, and combining corneal thickness and posterior curvature information is more effective than relying on only one of these measurements, especially in FFKC. Hwang et al. [48] have reported a better resolution in combining anterior curvature and asymmetry indices from Scheimpflug imaging [Pentacam HR (Oculus, Wetzlar, Germany)] with regional total thickness and epithelial thickness variability metrics from OCT (RTVue-100) for FFKC. Combining five Scheimpflug metrics provided better results, while combining 11 OCT thickness metrics yielded the best results from OCT. Moreover, combining 13 total Scheimpflug and OCT metrics gave the highest results. The most impactful variables included epithelial thickness variability and total focal corneal thickness variability from OCT, along with anterior curvature and topometric indices from Scheimpflug technology. Interestingly, no posterior corneal indices were significant in this model.

For the ANTERION (a swept-source system from Heidelberg Engineering GmbH, Heidelberg, Germany), curvature parameters of the anterior and posterior corneal surface have been studied. Saad et al. examined anterior and posterior curvature and thickness parameters. They reported an anterior curvature–derived parameter, the inferior-superior asymmetry value (I-S value) [39], yielded the highest AUROC in discriminating FFKC from normal eyes, followed by the magnitude of inferior decentration of posterior steepest keratometry [95].

For the CASIA SS-1000/CASIA2 (a swept-source system from Tomey Corporation, Aichi, Japan), posterior corneal elevation has also been studied in FFKC. According to Fukuda et al., posterior corneal elevation was determined to demonstrate the most reliable predictive accuracy in detecting FFKC [101]. Kitazawa et al. and Itoi et al. calculated and demonstrated that the ratio of the anterior corneal surface area to the posterior corneal surface area, derived from elevation maps obtained through OCT, was useful for discriminating FFKC cases from normal eyes, indicating that an imbalance in the anterior and posterior corneal surface areas may reflect early-stage keratoconus [102, 103]. Regarding quantitative Fourier harmonic analysis of corneal power distribution, Maeno et al. have reported that posterior irregular corneal astigmatism exhibits the highest discrimination ability among anterior or posterior components for FFKC [99]. Automated single parameters and newly computed indices using the CASIA have been combined to further improve this accuracy [104]. Shiga et al. have studied Fourier parameters with the CASIA as well, indicating the combination of the posterior corneal asymmetric component and central corneal thickness in FFKC discrimination [105]. Moreover, when the posterior corneal asymmetric component from anterior segment optical coherence tomography (AS-OCT) and the parameter SP-A1 from corneal biomechanical assessment (Corvis ST, Oculus Optikgeräte) were combined, it provided higher AUROC, although no significant differences in AUROC across diagnoses when comparing the use of each individual device versus their combined uses (integrated parameters versus AS-OCT, *P* = 0.081; integrated parameters versus Scheimpflug-based tonometer, *P* = 0.234).

Diagnosing FFKC by integrating biomechanical properties with morphology analysis has shown superiority compared to diagnosis based on a single device. Combining parameters obtained from multimodal imaging, such as Scheimpflug and OCT epithelial thickness mapping [48, 106], or tomographic and biomechanical assessments [35, 80] result in better detection compared to individual parameters. This indicates that corneal biomechanics can be a complementary tool for FFKC identification in addition to tomographic parameters. In fact, rather than a single individual Fourier posterior asymmetry component, enhanced subclinical ectasia detection capability by combining posterior Fourier indices and biomechanical properties might be possible.

**Fig. 2:**
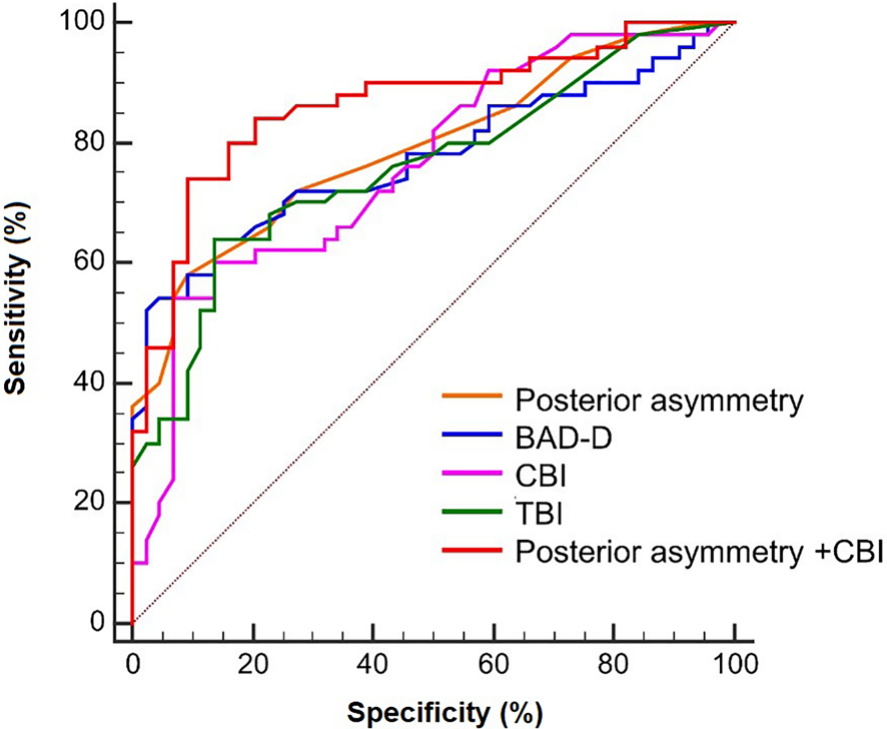
Receiver operating characteristic (ROC) curves of single and combined parameters for forme fruste keratoconus (FFKC) discriminating ability. The discriminating ability accuracies of the Fourier indices were assessed between FFKC and control. ROC curves for each Fourier index for the central 6 mm of the cornea, the asymmetry component of the anterior and the posterior cornea, CBI, TBI, BAD-D, and combined posterior asymmetry and CBI (combined metric) are shown. FFKC eyes had the highest area under the ROC curve (AUROC) values for the combined metric. CBI corneal biomechanical index; TBI, tomographic and biomechanical index; BAD-D, Belin/Ambrósio enhanced ectasia display total deviation

With approval from the Institutional Review Board/Ethics Committee of Osaka University Hospital (registration number: 09297–20), we re-examined our previously published AS-OCT from 50 FFKC eyes of 50 patients and 44 controls [99] alongside Scheimpflug-based tomography and biomechanical data obtained from the same patients using Pentacam HR and Corvis ST, respectively [99]. After determining BAD-D using Scheimpflug-based tomography (Pentacam HR), CBI and TBI using corneal biomechanical assessment (Corvis ST), and the posterior asymmetry component from the Fourier analysis with OCT (CASIA2), we compared the AUROC, sensitivity, and specificity for these four indices and variable combinations. Despite this, no individual parameter from Scheimpflug, OCT, or biomechanical assessment yielded an AUROC higher than 0.80 (Fig. 2). Fourier posterior asymmetry with OCT gave the highest AUROC (0.778) [99] (see Table 5). When parameters were combined using multiple regression analysis, the combination of posterior corneal asymmetry and CBI significantly improved the discrimination accuracy for FFKC (Fig. 2) (*P* = 0.024 between ROCs for the Fourier posterior asymmetry component and the combined metric). This yielded the highest AUROC (0.863) almost equivalent to other combined parameters by Shiga et al., and improved sensitivity compared to single parameter detection (Table 5). Further studies combining both devices may be conducted, as differences in sample size could be contributing to the variation in these results.

**Table 5:** Optimum cut-off values with the highest sensitivity and specificity

Parameters	FFKC vs. Normal			
	AUROC	Sensitivity	Specificity	**Cutoff**
Posterior asymmetry (D)	0.778	0.58	0.88	0.08
	*P* < 0.001			
CBI	0.762	0.54	0.93	0.46
	*P* < 0.001			
BAD-D	0.774	0.64	0.86	1.43
	*P* < 0.001			
TBI	0.759	0.64	0.86	0.25
	*P* < 0.001			
Posterior asymmetry + CBI	0.863	0.74	0.91	N/A
	*P* < 0.001			

*FFKC* = forme fruste keratoconus; *AUROC* = area under the receiver operating characteristic curve; *CBI* = Corvis biomechanical index; *BAD-D* = Belin/Ambrósio enhanced ectasia display total deviation; *TBI* = tomographic and biomechanical index

### OCT in the early diagnosis with corneal epithelial thickness mapping

Changes in total corneal thickness associated with keratoconus arise from alterations in both stromal and epithelial layers [107, 108]. In keratoconus, the epithelium exhibits thinning in the area overlying the cone, and this phenomenon has been confirmed through corneal epithelial mapping with OCT [109, 110]. This epithelial remodelling occurs in response to underlying stromal irregularities, with progressive stromal thinning contributing to the advancement of the disease. Recent progress have enabled thickness mapping of both epithelial and stromal thickness profiles [111–113].

Using RTVue, epithelial thickness mapping reportedly detect the initiating event of keratoconus that cannot be identified using topographic and tomographic analyses [109, 111, 114, 115]. Furthermore, more devices such as CASIA2 have recently been shown to effectively measure corneal layer thickness, providing valuable insights for detecting keratoconus [116]. Instruments such as ANTERION, MS-39, and Avanti also enable epithelial thickness measurements in eyes affected by keratoconus [110, 117].

Distinguishing FFKC from normal corneas warrants further study as a single corneal epithelial thickness parameter has proven inadequate for detecting the very early stages of keratoconus. To enhance diagnostic precision, efforts have been made to incorporate parameters from posterior cornea, corneal biomechanics or the standard deviation of corneal epithelial pattern [106, 114, 118]. Researchers have emphasized that combining multiple diagnostic modalities is critical for improving early detection. Additionally, custom-designed polarization-sensitive OCT based on swept-source OCT technology, has shown potential in visualizing the pathological changes in keratoconus including changes in the Bowman layer [119], and could be a valuable tool for identifying FFKC.

## Conclusions

As highlighted in this review, the criteria for FFKC, SKC and VAE-NT often differ between studies and there is a lack of consistency regarding the terminology of early keratoconus detection. As measurement principles differ depending on the technique used for keratoconus assessment, comprehensive metrics may be needed to reflect subtle anterior or posterior corneal changes and help identify eyes with very early ectasia.

In this review, we primarily utilized AUROC values as a standard measure of diagnostic accuracy to demonstrate the efficacy of various diagnostic tools. However, it should be noted that incorporating additional statistical measures, such as positive predictive value and negative predictive value, may provide a more comprehensive evaluation of the clinical utility of these diagnostic tools.

In the foreseeable future, techniques such as OCT, Scheimpflug, non-contact tonometry (Corvis ST) and genetic analysis will undoubtedly continue to play a key role in the early detection of keratoconus. The advancement of these technologies and the further development of other less well investigated techniques such as Brillouin light-scattering microscopy, ultra-high-resolution ultrasound, optical coherence elastography and atomic force microscopy may lead to the earlier detection of keratoconus. Although it is crucial to acknowledge that clinical experts have always, and will most likely, continue to play a pivotal role in decision-making for early keratoconus diagnosis, it is envisaged that data interpretation will likely be enhanced by AI.

## Data Availability

Not applicable.

## Abbreviations

A1L: Length at the first applanation
A2L: Length at the second applanation
A1T: Time to reach the first applanation
A2T: Time to reach the second applanation
A1V: Velocity at the first applanation
A2V: Velocity at the second applanation
ACE-NT: Asymmetric contralateral corneal ectasia with normal topography
AI: Artificial intelligence
AKC: Asymmetric keratoconus
ART: Ambrósio relational thickness
ARTh: Ambrósio relational thickness to the horizontal profile
ARTmax: Maximum Ambrósio relational thickness
AUROC: Area under the receiver operating characteristic curve
BAD: Belin/Ambrósio enhanced ectasia display
BAD-D: Belin/Ambrósio enhanced ectasia display total deviation
bIOP: Biomechanically adjusted intraocular pressure
CBI: Corneal biomechanical index
cCBI: CBI optimized for Chinese patients
CCT: Central corneal thickness
CDVA: Corrected distance visual acuity
CH: Corneal hysteresis
Corvis ST: Corneal visualization Scheimpflug tonometer
CRF: Corneal resistance factor
DA ratio: Deformation amplitude ratio
DA: Deformation amplitude
FFKC: Forme fruste keratoconus
H1: Height from lowest to highest point in peak 1
H2: Height from lowest to highest point in peak 2
IOP: Intraocular pressure
IR: Integrated radius
I-S value: Inferior–superior asymmetry value
KC: Keratoconus
KISA%: Keratoconus percentage
Kmax: Maximum keratometry
KSS: Keratoconus Severity Score
OCT: Optical coherence tomography
ORA: Ocular response analyser
p1 area: Upper 75% area of peak 1
Pr: Resultant pressure
ROC: Receiver operating characteristic curve
SKC: Subclinical keratoconus
SP-A1: Stiffness parameter at the first applanation
SSI: Stress-strain index
TBI: Tomographic and biomechanical index
VAE: Very asymmetric ectasia
VAE-NT: Very asymmetric ectasia where one eye has clinical keratoconus and the fellow eye appears topographically normal
VAE-NTT: Very asymmetric ectasia where one eye has clinical keratoconus and the fellow eye appears topographically and tomographically normal
